# Attention-Enhanced Semantic Segmentation for Substation Inspection Robot Navigation

**DOI:** 10.3390/s25196252

**Published:** 2025-10-09

**Authors:** Changqing Cai, Yongkang Yang, Kaiqiao Tian, Yuxin Yan, Kazuyuki Kobayashi, Ka C. Cheok

**Affiliations:** 1 College of Electrical and Information Engineering, Changchun Institute of Technology, 395 Kuan Ping Road, Changchun 130103, China; yangyongkang@stu.ccit.edu.cn (Y.Y.); yanyuxin@stu.ccit.edu.cn (Y.Y.); 2 Electrical and Computer Engineering, Oakland University, Rochester, MI 48309, USA; cheok@oakland.edu; 3 Department of Advanced Sciences, Hosei University, Tokyo 184-8584, Japan; ikko@hosei.ac.jp

**Keywords:** autonomous inspection robot, semantic segmentation, DeepLabV3+, attention mechanism, navigation line fitting, multimodal perception

## Abstract

Outdoor substations present complex conditions such as uneven terrain, strong illumination variations, and frequent occlusions, which pose significant challenges for autonomous robotic inspection. To address these issues, we develop an embedded inspection robot that integrates attention-enhanced semantic segmentation with GPS-assisted navigation for reliable operation. A lightweight DeepLabV3+ model is improved with ECA-SimAM and CBAM attention modules and further extended with a GPS-guided attention component that incorporates coarse location priors to refine feature focus and improve boundary recognition under challenging lighting and occlusion. The segmentation outputs are used to generate real-time road masks and navigation lines via center-of-mass and least-squares fitting, while RTK-GPS provides global positioning and triggers waypoint-based behaviors such as turning and stopping. Experimental results show that the proposed method achieves 85.26% mean IoU and 89.45% mean pixel accuracy, outperforming U-Net, PSPNet, HRNet, and standard DeepLabV3+. Deployed on an embedded platform and validated in real substations, the system demonstrates both robustness and scalability for practical infrastructure inspection tasks.

## 1. Introduction

Outdoor substations are critical components of power grids, but present complex environments characterized by uneven terrain, varying illumination, frequent occlusions, and strong electromagnetic interference. Reliable inspection is therefore essential for preventive maintenance and fault detection. Recent studies have addressed substation environmental monitoring, such as temperature–humidity regulation for equipment reliability and developed vision-based methods for enhancing low-illumination meter images to improve reading accuracy [1,2]. Traditional inspection methods, including manual patrols or fixed cameras, are labor-intensive, costly, and often insufficient in such challenging conditions. In addition to environmental monitoring and meter reading, recent research has also explored autonomous inspection technologies for substations, including resident UAV-based solutions for aerial monitoring and intelligent ground robots for automated equipment inspection [3,4].

Early autonomous inspection systems adopted magnetic guide tracks, RFID beacons, or 2D LiDAR paired with differential GPS for basic navigation [5,6]. While functional, these approaches suffer from high maintenance costs, poor adaptability, and limitations in dynamic or unstructured terrains challenges commonly found in substations [7].

With the rapid development of deep learning, mobile robots can now achieve scene-level semantic understanding without additional infrastructure [8,9,10]. Semantic segmentation enables direct extraction of drivable regions and environmental features from raw images, forming the basis for perception-driven navigation [11,12,13,14]. Architectures such as DeepLabV3+ and its attention-enhanced variants have shown strong performance in outdoor scene interpretation [15,16,17]. More recently, transformer-based approaches and multimodal fusion networks have further advanced semantic segmentation by capturing long-range dependencies and integrating cross-sensor information [18,19,20].

However, vision-only systems are still prone to degraded performance under adverse conditions such as strong illumination, shadows, and occlusion scenarios frequently encountered in substations. To mitigate these challenges, recent research has introduced multimodal fusion strategies, for example, combining visual perception with auxiliary signals such as GPS or LiDAR and developing location-aware attention mechanisms to incorporate positional priors into feature extraction [21,22]. These approaches demonstrate the potential of multimodal perception, but many remain computationally expensive and have seen limited validation in realistic substation inspection tasks.

In this work, we propose an attention-enhanced semantic navigation framework tailored for substation inspection robots.

A lightweight DeepLabV3+ backbone is augmented with ECA-SimAM and CBAM modules to refine feature extraction under challenging lighting and occlusion. In addition, a GPS-guided attention component provides coarse positional priors to further stabilize segmentation performance, while GPS feedback is used at the system level for waypoint-based behavior control. To support training, we constructed and annotated a custom dataset of substation road scenes under diverse conditions. The proposed framework is thoroughly validated through ablation studies, baseline comparisons, and real-world substation experiments, demonstrating both improved segmentation accuracy and reliable autonomous navigation.

## 2. Methodology

### 2.1. System Overview

**Figure 1:**
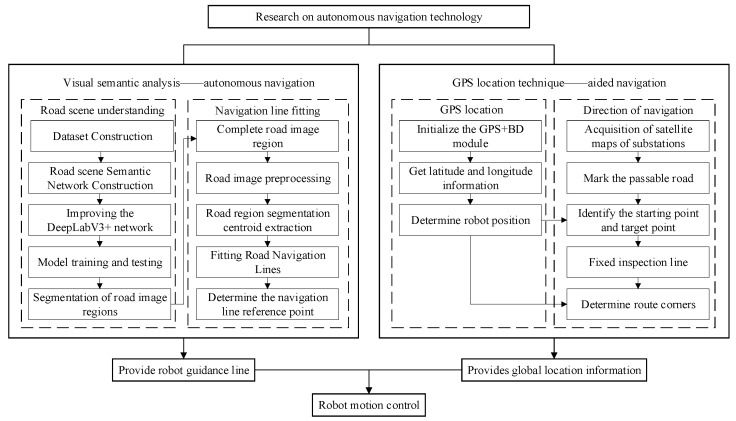
System architecture integrating semantic perception and GPS localization.

The proposed autonomous navigation framework integrates vision-based local path planning with RTK-GPS-assisted global localization. This combination enables robust inspection in complex substation environments. As shown in Figure 1, the robot first captures real-time road images through an onboard RGB camera. These images are processed by an improved DeepLabV3+ model with attention mechanisms, which produce binary road masks. A navigation line is then fitted to the segmented road regions using center-of-mass and least-squares methods. This line provides the robot’s direction of travel.

To train the model, a custom dataset was collected and manually annotated into four semantic categories: main road, branch road, stone, and background. The dataset includes diverse conditions such as shadows, occlusions, and illumination variations. This ensures that the trained network is robust under realistic substation environments.

During deployment, the predicted road masks are further divided longitudinally, and navigation lines are fitted to guide the robot’s movement. RTK-GPS serves as a complementary module. It provides coarse global positioning that is aligned with a preprocessed binarized satellite map, which separates drivable from non-drivable areas. GPS feedback is also used for waypoint-based behavior control, such as triggering turns or stops at intersections. By combining high-precision local perception with GPS-based global cues, the system achieves precise and adaptive autonomous navigation in substations.

### 2.2. Hardware Architecture

**Figure 2:**
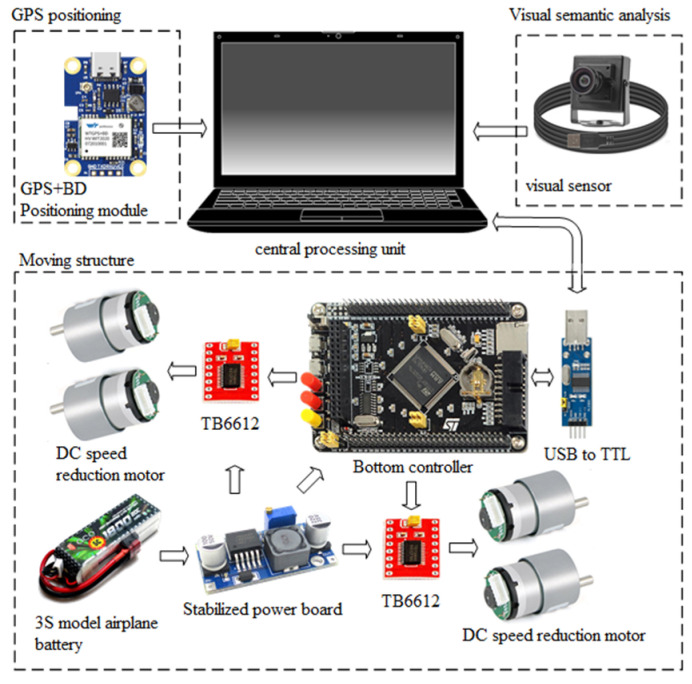
Hardware connection block diagram.

The mobile inspection platform is composed of three primary subsystems: the visual semantic analysis module, the GPS localization module, and the motion control module. As shown in Figure 2, a high-performance industrial PC serves as the central processing unit, integrating a camera for image acquisition and a GPS module for position sensing.

**Figure 3:**
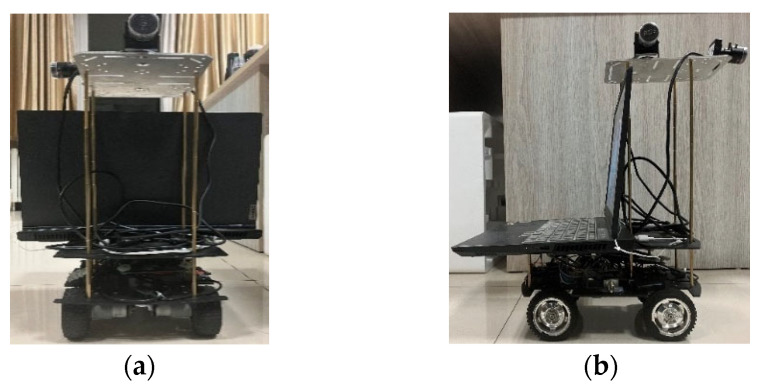
Physical drawing of the robot’s mobile mechanism: (**a**) front view; (**b**) side view.

The motion control system is driven by an STM32F103ZET6 microcontroller, which uses a position-based PID algorithm to regulate the speeds of four DC geared motors via a motor driver module. Real-time motor speed feedback is obtained through Hall-effect encoders. The mechanical platform features a four-wheel differential drive configuration powered by MG513 DC geared motors, providing sufficient torque for outdoor traversal. The physical structure of the robot is illustrated in Figure 3.

The vision module includes a high-resolution camera (3840 × 2160, MJPG format, Shenzhen Minrray Industry Co., Ltd., Shenzhen, Guangdong, China), connected to a PC equipped with an AMD Ryzen 7 4800H CPU and an NVIDIA GTX 1650Ti GPU. This setup supports the deployment of a deep learning-based semantic segmentation network that enables real-time scene understanding for the robot.

### 2.3. Software Architecture

Training Environment:

Model training is conducted using the PyTorch framework (version 2.1.0) on an Ubuntu 18.04 system. The training environment is optimized for large-scale dataset processing and includes CUDA 10.1 and cuDNN 7.6.5. The workstation is configured with 43 GB of RAM and an 11 GB GPU memory.

Runtime Environment:

For real-time deployment, the segmentation model runs under Windows 10 using the same PyTorch framework. The runtime PC is equipped with an AMD Ryzen 7 4800H processor, 16 GB RAM, 4 GB GPU memory, and the same CUDA/cuDNN versions.

Motor Control Environment:

The STM32 firmware for motor control is developed using Keil MDK-ARM, a popular integrated development environment (IDE) for embedded systems.

### 2.4. Dataset Collection and Annotation

To support the training of the semantic segmentation model, a custom dataset of substation road scenes was collected under diverse environmental conditions, including varying illumination, shadows, and occlusions. In total, 1200 RGB images were captured at a resolution of 1920 × 1080 pixels using the onboard camera. Each image was manually annotated into four semantic categories: main road, branch road, stone, and background. Annotation was performed using the LabelMe tool, and all masks were cross-checked by two annotators to ensure consistency and accuracy.

The dataset was divided into 70% training (840 images), 15% validation (180 images), and 15% testing (180 images). The approximate distribution of annotated objects is as follows: main road (45%), branch road (25%), stone (10%), and background (20%). This balanced representation ensures that the network learns both major drivable regions and challenging minor categories.

In addition to visual data, GPS logs were simultaneously recorded using an RTK-enabled GPS module (nominal horizontal accuracy ±2 cm at 10 Hz). GPS readings were synchronized with the camera stream via timestamp alignment, providing coarse location priors for the GPS-guided attention mechanism and waypoint-based navigation experiments.

### 2.5. Semantic Segmentation Network Design

To enable accurate substation road scene understanding, this section presents an enhanced semantic segmentation architecture based on DeepLabV3+. Classical architectures such as U-Net [23], PSPNet [24], and HRNet [25] have been widely adopted as baseline methods in scene segmentation, but they often struggle with blurred boundaries, occlusions, and illumination variations commonly encountered in substations. To address these challenges, attention mechanisms are introduced into the proposed DeepLabV3+ framework to improve feature extraction and boundary recognition under such adverse conditions. The model is further evaluated through ablation studies and benchmarked against both these baseline networks and other state-of-the-art methods.

#### 2.5.1. DeepLabV3+ Network Architecture

**Figure 4:**
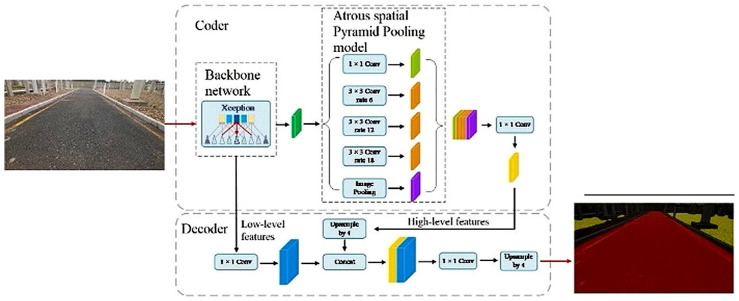
Classical DeepLabV3+ semantic segmentation model.

The DeepLabV3+ model consists of an encoder–decoder structure (Figure 4). The encoder includes a backbone feature extractor and an Atrous Spatial Pyramid Pooling (ASPP) module. The decoder refines the segmentation output by combining high-level semantic and low-level spatial features through up sampling operations.

#### 2.5.2. Backbone

**Figure 5:**
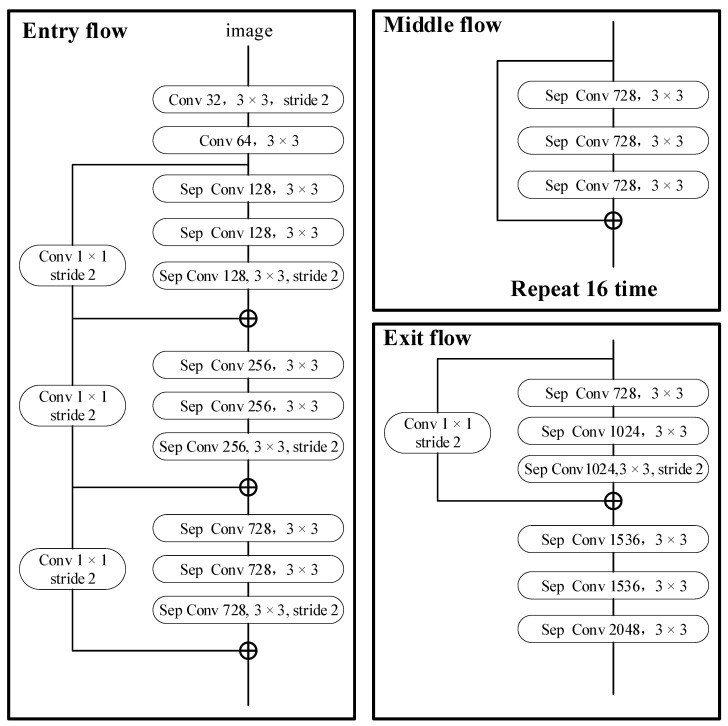
Xception network structure.

The backbone network uses an improved Xception architecture consisting of Entry flow, Middle flow, and Exit flow modules. Each max-pooling layer is replaced with a depthwise separable convolution with stride 2, followed by Batch Normalization (BN) and ReLU activations to improve training stability. The Middle flow is extended to 16 repeated blocks for deeper feature representation. The complete architecture contains 65 convolutional layers, as shown in Figure 5.

#### 2.5.3. Depthwise Separable Convolution

**Figure 6:**
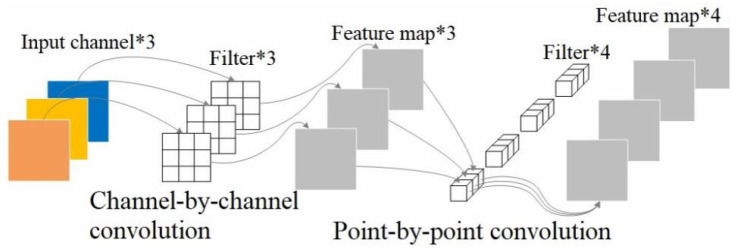
Depthwise separable convolution structure.

Depthwise separable convolution factorizes standard convolution into channel-wise and point-wise operations, reducing computation while preserving feature representation [26]. This decomposition enables efficient multi-scale learning. The structure is illustrated in Figure 6.

#### 2.5.4. ASSP Module

ASPP (Atrous Spatial Pyramid Pooling) enhances the receptive field by performing dilated convolution at multiple sampling rates. The effective kernel size is given by:

(1) $$y\left[i\right]=\underset{k}{\sum }x\left[i+rk\right]\omega \left[k\right]$$

(2) $$k_{e}=k+\left(k-1\right)\left(r-1\right)$$

where r is the dilation rate, k the filter size, and $k_{e}$ the expanded receptive field. This enables robust segmentation of objects at different scales.

#### 2.5.5. Attention Mechanisms

To improve boundary detection and reduce the effects of illumination and occlusion, attention modules are integrated. Three types are employed: ECA (Efficient Channel Attention) [27], SimAM (Simple Attention Module) [28], and CBAM (Convolutional Block Attention Module) [29].

**Figure 7:**
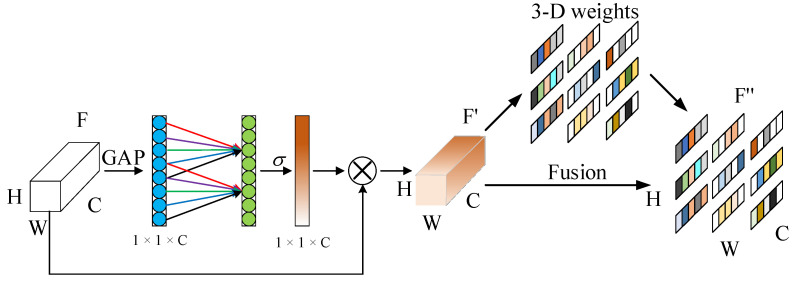
Structure of the ECA-SimAM attention network, where blue and green circles denote input and output feature nodes, red and blue lines indicate weighted connections for channel attention, the orange bar and block represent intermediate feature maps, and the multicolored stripes correspond to refined feature representations across different channels.

By introducing the attention mechanism into the DeepLabV3+ model, the image information is acquired, the edge features are refined, and the feature extraction ability of the model is improved. The ECA-SimAM attention module is composed of the serial connection between the ECA module and the SimAM module. As shown in Figure 7, this structure further enhances the feature extraction capability without increasing the number of parameters. The feature image F (H × W × C) is compressed into a one-dimensional feature vector via global average pooling. This vector is processed through a 1D convolution and Sigmoid activation to produce channel attention weights. These are multiplied with the input features to generate F’. A SimAM module then uses an energy function to estimate spatial importance and produce a refined feature map F”.

Meanwhile, the CBAM module is introduced into the ASPP component of the encoder. It further calibrates feature maps along both spatial and channel dimensions, enhancing the model’s ability to capture fine-grained semantic details. These attention integrations collectively boost segmentation performance in complex environments such as substations.

1. GPS-Based Attention Mechanism

To leverage RTK-GPS for location-aware segmentation, we propose a GPS-based attention module fused into the existing attention pipeline. GPS coordinates (latitude, longitude) are embedded into a low-dimensional vector via a fully connected layer, then concatenated with feature maps before attention computation. This modulates weights in CBAM, prioritizing features relevant to the current position (e.g., emphasizing branch roads at known junctions). The modulation is defined as:

(3) $$A_{gps}=\sigma \left(W_{gps}\left[F;E_{gps}\right]\right)$$

where F is the input feature, $E_{gps}$ is the GPS embedding, $W_{gps}$ is a learnable weight, and σ is sigmoid. This adds minimal parameters (~0.5% increase) but enhances adaptability in large-scale substations by incorporating global priors into local attention.

### 2.6. GPS-Assisted Path Planning

In this section, we describe a GPS-assisted visual navigation framework that integrates satellite-based localization with semantic perception for substation inspection robots. The goal is to enable robust and autonomous traversal of complex outdoor environments by combining global positioning information with local visual cues.

#### 2.6.1. Satellite Map Alignment and Drivable Region Extraction

To support high-level path planning, we first acquired a high-resolution RGB satellite image of the substation. A WTGPS + BD dual-mode positioning module provides real-time geographic coordinates, which are aligned to the satellite image through coordinate transformation. This mapping allows us to localize the robot within the satellite map.

**Figure 8:**
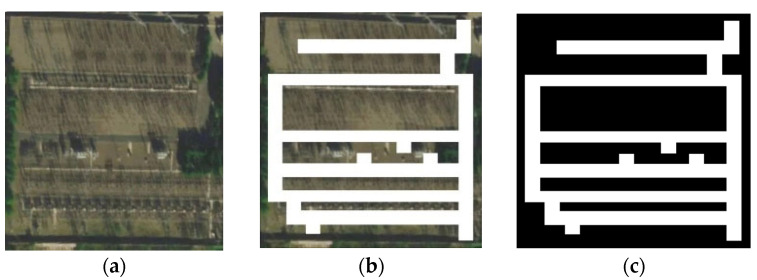
Preprocessing of satellite maps: (**a**) Substation RGB satellite map; (**b**) Marked Road; (**c**) binarized image.

The image is then manually annotated to highlight primary substation roads, after which it is binarized to segment drivable (white) and non-drivable (black) areas. As shown in Figure 8, the preprocessing steps include: (a) capturing the RGB satellite map, (b) marking roads, and (c) generating the binary mask. This process yields a global navigation map that simplifies subsequent path planning.

Navigable areas are extracted by binarizing the satellite image into drivable (white) and non-drivable (black) regions. This segmentation provides a global navigation map that distinguishes main roads and surrounding obstacles. Figure 8b shows the preprocessing results. To extract navigable areas, a binary mask is generated by segmenting road pixels (white) from non-road regions (black), simplifying path computation (Figure 8c).

Since lens distortion affects the accuracy of image-based navigation, Zhang’s calibration method is applied. A total of 60 checkerboard images is captured, and 49 valid samples are used to compute the intrinsic matrix and distortion coefficients. These parameters are obtained using MATLAB’s calibration toolbox (version R2024a) and improve the projection accuracy from 3D world coordinates to 2D image space. This correction is essential for precise navigation overlay and visual path estimation.

#### 2.6.2. Vision-Based Path Estimation and Tracking

For local navigation, the robot uses an onboard RGB camera to capture real-time road scenes. An improved DeepLabV3+ network segments drivable areas, from which a navigation line is fitted using center-of-mass and least-squares methods. The robot then calculates lateral deviation and heading angle offset from this line.

#### 2.6.3. GPS-Guided Waypoint Switching and Behavior Control

Predefined waypoints such as road intersections or target locations are embedded in the satellite map. When GPS indicates the robot is near a waypoint, it triggers behavior switching actions—such as turning, stopping, or initiating detailed inspections.

**Figure 9:**
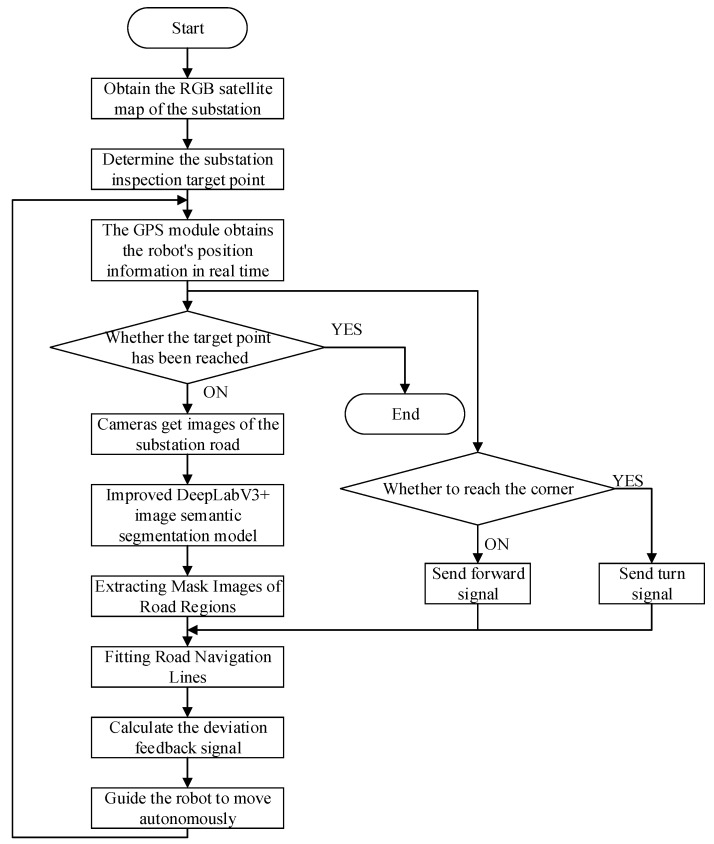
Flowchart of GPS-assisted visual navigation.

Figure 9 illustrates the hybrid navigation process, which consists of the following steps:

1. Define a fixed route by selecting target points on the satellite map.
2. Continuously monitor the robot’s position using real-time GPS coordinates.
3. Acquire forward-facing road images using the onboard camera.
4. Apply the improved DeepLabV3+ model to segment drivable road regions.
5. Generate a binary mask representing navigable areas.
6. Fit a navigation line using center-of-mass and least-squares techniques.
7. Compute the lateral deviation and heading angle with respect to the image center.
8. Issue forward motion or steering commands based on current deviation and robot status.

This hybrid system enables accurate path-following, real-time trajectory correction, and adaptive behavior switching at key waypoints, ensuring robust autonomous traversal of complex substation environments.

## 3. Experimental Results

### 3.1. Semantic Segmentation Performance

To evaluate the performance of the proposed segmentation network, we conducted a series of quantitative and qualitative experiments. The improved DeepLabV3+ model was compared against several baseline architectures, including U-Net, PSPNet, HRNet, and the original DeepLabV3+. All models were trained using the same substation road image dataset, under identical hyperparameter settings: a batch size of 8, 1000 training epochs, and the Adam optimizer. The configurations for each model are as follows:

1. U-Net: ResNet-50, learning rate = 0.0001.
2. PSPNet: ResNet-50, learning rate = 0.0005.
3. HRNet: HRNetv2-w18, learning rate = 0.0005.
4. DeepLabV3+: Xception, learning rate = 0.0003.
5. Improved DeepLabV3+: Improved Xception, learning rate = 0.0003.

Table 1 summarizes the evaluation metrics used to assess model performance, specifically the mean Intersection-over-Union ($e_{\mathrm{mIoU}}$) and mean Pixel Accuracy ($e_{\mathrm{mPA}}$):

The improved DeepLabV3+ model achieved the highest accuracy among all evaluated networks, with a 3.22% improvement in $e_{\mathrm{mIoU}}$ and a 2.99% gain in $e_{\mathrm{mPA}}$ over the baseline DeepLabV3+. These results confirm that incorporating attention modules into the backbone and ASPP components significantly enhances segmentation performance in complex outdoor scenes.

To further validate the benefits of attention modules, we performed ablation studies. Table 2 presents the results of selectively enabling different attention mechanisms. The combination of ECA and SimAM yielded the best performance, indicating that spatial and channel-wise refinement improves the model’s ability to distinguish road boundaries and small features. Incorporating GPS-based attention further boosts $e_{\mathrm{mIoU}}$ by 1.5% in location-varying tests, confirming its value in outdoor scenarios.

**Figure 10:**
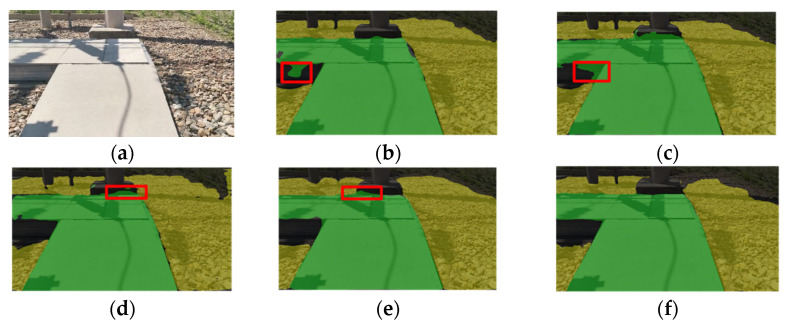
Segmentation Results on a Branch Corner Scene (**a**) Original picture; (**b**) U-Net; (**c**) PSPNet; (**d**) HRNet; (**e**) DeepLabV3+; (**f**) Improved DeepLabV3+.

In addition to quantitative analysis, visual results are shown in Figure 10. These demonstrate that the improved model produces smoother segmentation contours, better preserves road continuity under shadows and occlusions, and eliminates false positives often present in other networks.

Moreover, inference efficiency was measured to assess real-time feasibility. On an NVIDIA GTX 1650Ti, the improved model processes frame at ~31.8 MS per image, enabling near-real-time performance. Despite added attention modules, the model size only increased marginally, confirming the efficiency of the design.

To ensure a fair comparison, all baseline models (U-Net, PSPNet, HRNet, and standard DeepLabV3+) were trained and evaluated on the same custom dataset with identical training/validation/test splits. Input images were resized to 1920 × 1080, normalized to [0, 1], and data augmentation (random cropping, flipping, and illumination variation) was applied consistently across all models. Training hyperparameters such as learning rate, optimizer (Adam, lr = 1 × 10^−4^), batch size, and number of epochs were kept the same for all networks, ensuring input consistency and comparability of results.

These comprehensive results demonstrate that the proposed enhancements improve segmentation quality and maintain practical inference speed, providing a reliable foundation for autonomous robot navigation in substations.

### 3.2. GPS-Based Navigation Performance

To demonstrate the effectiveness of GPS-assisted navigation, a waypoint-based experiment was conducted in a real-world substation setting. Several key points—such as intersections and destination zones—were marked in GPS coordinates. During the experiment, the robot utilized real-time RTK-GPS to determine its current location and trigger behavior switching when approaching predefined waypoints.

The system successfully completed five separate navigation routes; each composed of 3–5 waypoints. The average GPS localization error was within 0.35 m, and the waypoint triggering accuracy exceeded 96.8%. Table 3 shows an example path with GPS logs overlaid on the satellite map.

These results show that although visual segmentation remains the primary driver for local motion planning, GPS contributes to high-level path alignment and behavior control, especially at junctions or transitions between mission phases.

These comprehensive results demonstrate that the proposed enhancements improve segmentation quality and maintain practical inference speed, providing a reliable foundation for autonomous robot navigation in substations. The hybrid use of GPS and vision ensures robust, scalable performance across structured and semi-structured environments.

## 4. Conclusions

This paper presents an attention-enhanced visual navigation framework designed to address the challenges of autonomous inspection in complex substation environments. A lightweight DeepLabV3+ backbone was improved by embedding ECA-SimAM modules into the encoder and CBAM into the ASPP component, significantly enhancing both spatial and channel-wise feature representation. These improvements enable more accurate recognition of fine-grained edges and road boundaries under adverse lighting and occlusion.

To overcome the limitations of the classical DeepLabV3+ in road segmentation, the framework incorporates two major enhancements. First, the Xception backbone is optimized by embedding ECA-SimAM attention modules after each depthwise separable convolution. This modification enhances both spatial and channel-wise feature representation, resulting in improved object recognition and localization. Second, attention mechanisms are introduced into the Atrous Spatial Pyramid Pooling (ASPP) module, enabling more accurate detection of fine-grained edges and road boundaries—critical for navigating complex substation road environments. Third, a novel GPS-based attention mechanism fuses location data into the attention pipeline, making segmentation location-aware and further improving robustness in position-dependent scenarios.

Experimental results validate the effectiveness of the proposed improvements. The enhanced DeepLabV3+ model outperforms baseline networks such as U-Net, PSPNet, HRNet, and the original DeepLabV3+, achieving an $e_{\mathrm{mIoU}}$ of 85.26% and $e_{\mathrm{mPA}}$ of 89.45%. (with GPS-based attention pushing $e_{\mathrm{mIoU}}$ to 86.76% in ablation tests).

In addition, a hybrid vision-GPS navigation scheme is implemented, wherein the robot performs global path planning using satellite maps while adjusting its trajectory in real time based on semantic segmentation and GPS feedback. When the robot approaches key waypoints—such as corners or intersections, behavioral commands like turning or stopping are automatically triggered.

By combining deep semantic understanding with precise global positioning, enhanced by GPS-guided attention, this framework delivers a scalable and high-performance solution for autonomous substation inspection. It lays a solid foundation for future development of intelligent, reliable, and fully autonomous robotic systems in power infrastructure maintenance.

## 5. Future Work

Future work will focus on enhancing the robustness and scalability of the proposed system through several avenues. One direction is the integration of multimodal sensor data—including LiDAR, infrared thermal imaging, and depth or stereo cameras—to improve perception under challenging conditions such as poor lighting, rain, or occlusion. In parallel, robustness under adverse environmental conditions (e.g., heavy shadows, dynamic obstacles, or extreme weather) will be systematically investigated to identify failure cases and develop adaptive strategies. In addition, the robustness of the framework under weak or degraded GPS signals will be explored, with visual–inertial odometry or LiDAR-based localization considered as complementary solutions to maintain reliable navigation. Another promising area involves collaborative multi-robot navigation, which would enable robots to share information and coordinate tasks to increase inspection efficiency. Optimization of the deployment pipeline will also be a priority, with research into model compression techniques (such as pruning and quantization), accelerated edge inference, and lightweight network architectures to ensure real-time processing on resource-constrained platforms. Additionally, energy-aware motion planning and intelligent battery management will be explored to extend autonomous operational time. Finally, seamless integration with substation management systems, such as SCADA or digital twin platforms, will enable synchronized data reporting, real-time anomaly detection, and intelligent task scheduling, pushing the system closer to large-scale deployment. Also, advanced GPS-based attention variants, such as transformer-integrated fusion, will be explored for even finer multimodal adaptation.

## Figures and Tables

**Table 1:** Evaluation of segmentation accuracy across models.

Network Model	Core Network	$e_{mIoU}$	$e_{mPA}$
U-Net	ResNet-50	74.88%	81.07%
PSPNet	ResNet-50	79.53%	84.13%
HRNet	HRNetv2-w18	80.81%	85.93%
DeepLabV3+	Xception	82.04%	86.46%
Improved DeepLabV3+	Improved Xception	85.26%	89.45%

**Table 2:** Ablation study of attention modules.

Configuration	$e_{mIoU}$	$e_{mPA}$
Baseline (no attention)	82.04%	82.04%
+ ECA only	83.21%	83.21%
+ SimAM only	83.75%	83.75%
+ CBAM in ASPP	84.50%	84.50%
+ ECA + SimAM (proposed)	85.26%	85.26%
+ GPS-Based Attention	86.76%	90.95%

**Table 3:** Example GPS-assisted navigation path overlaid on satellite imagery.

Metric	Value
Average GPS error (RTK)	0.35 m
Waypoint detection accuracy	96.8%
Navigation completion rate	100% (5/5)
Average completion time	~90 s per route
Path deviation from fitted centerline	0.28 m
Robustness under shadows/occlusions	>92% route continuity

## Data Availability

The data presented in this study is available on request from the corresponding author.

## Abbreviations

The following abbreviations are used in this manuscript:

RTK-GPS	Real-Time Kinematic Global Positioning System
e_mIoU	Effective Mean Intersection-over-Union
e_mPA	Effective Mean Pixel Accuracy
ASPP	Atrous Spatial Pyramid Pooling
ECA	Efficient Channel Attention
SimAM	Simple Attention Module
